# To the Public

**Published:** 1883-01

**Authors:** 


### TO THE PUBLIC.

We want ten thousand new subscriptions at One Dollar each. We therefore issue many thousands of extra copies, which we send gratuitously to every section of the United States and Canada ; and we only ask those into whose hands a copy may fall, to give it a fair and impartial perusal and then decide if it is possible to make a better investment with $1.

The Journal of Health has now commenced its thirtieth year. It is the oldest health publication in the world, and we can say without contradiction that it is the most popular, and the most extensively quoted by other publications.

The Journal is essentially a publication for the people. It aims to convince—particularly the young—that there can be no assured success in the active affairs of life, and but little happiness, without sound health; that in a vast majority of cases of feeble health in adults, the causes of their troubles may be traced to imprudences committed in youth, through ignorance. We desire most to attract the attention of the young to the pages of the Journal. We know, however, that a publication devoted exclusively to discussions of health subjects would not be so attractive to these, or even to older persons, as one that also contained miscellaneous articles of interest to all, and therefore a portion of the Journal is devoted to such articles. Our plan has always been to employ plain language, and to use as few words as possible to express our meaning; and to this in a great measure we attribute the popularity of the JOURNAL: still another point of the greatest importance that parents should always have in view in the selection of reading for their children is the general tone of the publication.

There is a class of literature that is very demoralizing to the young, and whose effects are far-reaching and deplorable. The taste for this sort of reading is acquired through the facilities afforded at the present time of introducing it into almost every family.

The prime object of printing miscellaneous articles of interest in the JOURNAL is to counteract, as far as possible, the evil influence of bad books, by showing that a publication may be instructive and even attractive without being demoralizing in its tendencies.

EVERY NEW SUBSCRIBER WHO SENDS US ONE DOLLAR WILL RECEIVE THE JOURNAL OF HEALTH ONE YEAR. ALL PUBLIC INSTITUTIONS, SCHOOLS, SOCIETIES, ASSOCIATIONS, LIBRARIES, CLUBS, READING ROOMS or ORGANIZATIONS whatever—not already subscribers—that will send us 50 CENTS—which is barely the cost of the paper before it is printed—will receive the JOURNAL OF HEALTH ONE YEAR. Any Postmaster who will send us $2.00 with the names of two yearly subscribers, will receive a copy of the JOURNAL one year FREE. We are not aware that we have ever yet lost a dollar sent to us through the mails, and we do not think it necessary to register a letter containing so small a sum. Fractions of one dollar may be sent to us in United States postage stamps; we find the one-cent stamps most convenient and would prefer them.

Canadian subscribers may send Canada money; but we could not use Canadian postage stamps, as they could not use those of the United States. We advise new subscribers to keep this January number, if they desire to have the numbers for 1883 complete, for we may not be able to replace it. If the 12 numbers are sent to us at the end of the year, with 75 cents, we will have them bound in one volume and return postage paid. Address, HALL'S JOURNAL OF HEALTH, No. 135 Eighth street, New York.

WHEN, says Dr. Squib, the fixed stopper of a glass bottle resists all management—such as warming the neck with a cloth wet with warm water, by tapping and by the wrench, or by all these in combination—there is another means which will almost always succeed. Let the bottle be inverted, so as to stand on the stopper in a vessel of water so filled that the water reaches up to the shoulder of the bottle, but not to the label. Two or three nights of this treatment may be required sometimes before the stopper will yield.

